# Encapsulation of ultrafine metal-oxide nanoparticles within mesopores for biomass-derived catalytic applications†
†Electronic supplementary information (ESI) available: Experimental section, additional characterization and reaction results. See DOI: 10.1039/c7sc04724j


**DOI:** 10.1039/c7sc04724j

**Published:** 2018-01-04

**Authors:** Ruiqi Fang, Panliang Tian, Xianfeng Yang, Rafael Luque, Yingwei Li

**Affiliations:** a State Key Laboratory of Pulp and Paper Engineering , School of Chemistry and Chemical Engineering , South China University of Technology , Guangzhou 510640 , China . Email: liyw@scut.edu.cn; b Analytical and Testing Centre , South China University of Technology , Guangzhou 510640 , China; c Departamento de Química Orgánica , Universidad de Córdoba , Edif. Marie Curie, Ctra Nnal IVa, Km 396 , E14014 , Córdoba , Spain . Email: q62alsor@uco.es

## Abstract

The development of efficient encapsulation strategies has attracted intense interest for preparing highly active and stable heterogeneous metal catalysts. However, issues related to low loadings, costly precursors and complex synthesis processes restrict their potential applications. Herein, we report a novel and general strategy to encapsulate various ultrafine metal-oxides nanoparticles (NPs) into the mesoporous KIT-6. The synthesis is facile, which only involves self-assembly of a metal–organic framework (MOF) precursor in the silica mesopores and a subsequent calcination process to transform the MOF into metal-oxide NPs. After the controlled calcination, the metal-oxide NPs produced from MOF decomposition are exclusively confined and uniformly distributed in the mesopores of KIT-6 with high metal loadings. Benefitting from the encapsulation effects, as-synthesized Co@KIT-6 materials exhibit superior catalytic activity and recycling stability in biomass-derived HMF oxidation under mild reaction conditions.

## Abstract 2

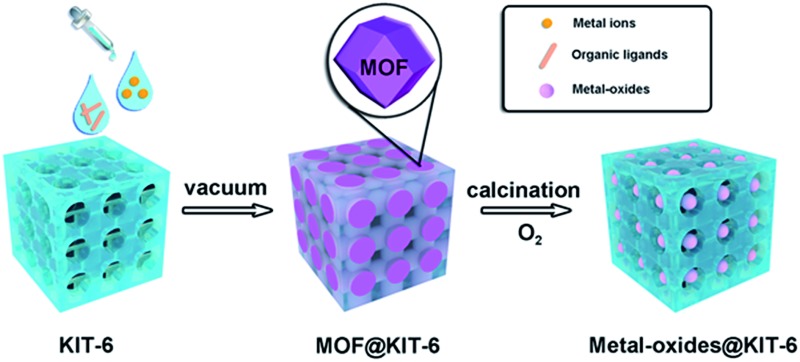
A versatile strategy using MOFs as precursors to encapsulate ultrasmall non-noble metal-oxides nanoparticles in the mesopores of KIT-6 is developed.

## Introduction

Declining fossil energies together with the continuous increase in environmental problems calls for more environmentally friendly processes towards the sustainable utilization of renewable raw materials.^1^–^5^ As compared to fossil resources, biomass, the only renewable carbon resource, exhibits great potential in producing a large number of value-added chemicals and fuels.^6^–^8^ Among various reaction routes and chemical intermediates in biomass transformations, biomass-derived furanic molecules (*e.g.*, 5-hydroxymethylfurfural (HMF) and furfural) have attracted significant attention in recent years as the bridge linking raw biomass and value-added chemicals.^9^–^11^

As a representative biomass-based platform molecule, HMF can be directly obtained from the acidic dehydration of hexoses (from cellulose) and has been widely used in the bio-industry to produce a variety of value-added chemicals (*e.g.*, 2,5-furandicarboxylic acid (FDCA), and 2,5-diformylfuran).^12^,^13^ Amongst these chemical products, FDCA is a very important industrial intermediate widely utilized in producing pharmaceuticals, antifungal agents, furanic biopolymers and furan-based resins as the alternative to petroleum-derived terephthalic acid.^14^,^15^ FDCA may be directly synthesized through oxidation of HMF using homogeneous catalytic systems. Unfortunately, these oxidation systems, that include Pd salts, KMnO_4_ and Co(OAc)_2_/HBr/Mn(OAc)_2_, are mostly toxic, corrosive and difficult to be recycled, far from meeting current strict environmental regulations and scale-up production needs.^16^,^17^ To overcome these shortcomings, heterogeneous catalysts have been recently employed in HMF oxidation. A variety of supported metal catalysts have been developed for HMF oxidation based on graphene oxides, active carbons, metal–organic frameworks (MOFs) and zeolites as catalysts and supports. However, metal nanoparticles (NPs) on these supports prepared by the traditional recipes, such as ion exchange or wetness impregnation, tended to aggregate on the catalyst surface, resulting in an unsatisfying activity and recyclability.^18^–^20^

Metal confinement in porous materials has been demonstrated as an efficient strategy to enhance the dispersion and stability of metal NPs. In this regard, mesoporous and zeolite-like materials (*e.g.*, mesoporous silicas, zeolites) have drawn growing attention as ideal hosts for the confinement of metal NPs, due to their well-defined channels, large porosity and good thermal/chemical stability.^21^,^22^ Accordingly, a few novel strategies have been explored to encapsulate metal NPs into the layers or pores of mesoporous silicas and zeolites, as well as other hard templates, mostly focusing on the confinement of noble metals owing to their nonmagnetic properties and chemical inertness throughout the synthesis processes.^23^–^29^ For example, Liu *et al.* demonstrated a swelling-calcination method to obtain encapsulated 3D Pt@MCM-22 materials by introducing Pt NPs during the growth of MCM-22.^30^ Dai *et al.* reported a recrystallization method to confine a series of Pt based NPs within a silicalite-1 matrix.^31^ Iglesia *et al.* have recently developed a ligand-stabilized method to encapsulate a series of noble metal NPs (*e.g.*, Pt, Ir, Rh, Pd and Ag) into different zeolites.^32^ Yu *et al.* and Xiao *et al.* reported an *in situ* confinement strategy for encapsulating Pd NPs into zeolites.^33^,^34^ Despite the success of confining well dispersed metal NPs, it is very difficult to simultaneously achieve a high metal loading (normally < 2 wt%) and high dispersion upon confinement. On the other hand, the costly metal precursors or complex synthesis processes largely restrict their large-scale applications.^35^–^38^ Therefore, the development of a facile and efficient strategy for the encapsulation of ultrafine metal NPs at a high loading still remains a great challenge in heterogeneous catalysis.

Herein, we report a novel and versatile strategy for the encapsulation of various ultrafine metal-oxides within mesoporous silicates at a high metal loading amount of up to 13.6 wt%. The synthesis is facile, which only involves self-assembly of a MOF precursor in the pores of mesoporous silica and a subsequent calcination process to convert the MOF into metal-oxide NPs. KIT-6, a typical mesoporous silica, has been used as a representative substrate for this synthesis approach. The obtained Co@KIT-6 nanocomposites exhibit superior catalytic activity and stability in HMF oxidation, achieving a complete conversion and >99% FDCA selectivity with turnover frequency (TOF) values as high as 150 h^–1^ at 80 °C and 0.1 MPa air using water as a solvent. The observed TOF is 3–150 times greater than those of the previously reported heterogeneous noble-metal catalysts under milder, or at least comparable conditions.

## Results and discussion

**Scheme 1:**
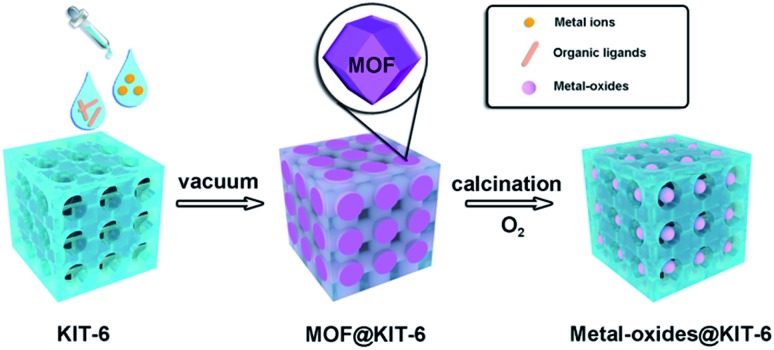
Synthesis route of metal-oxides@KIT-6.

The typical synthesis route of metal-oxides@KIT-6 is illustrated in Scheme 1. Taking the encapsulation of cobalt oxide NPs as an example, a methanol solution containing Co(NO_3_)_2_ and KIT-6 was first treated with 2-methylimidazole to form ZIF-67@KIT-6 under vacuum. The transformations of KIT-6 structures during the synthesis process were monitored by powder X-ray diffraction (XRD) (Fig. S1†). Low-angle XRD patterns indicated that the mesoporous structure exhibited the typical *Ia*3*d* symmetry, well preserved after MOF encapsulation. The wide diffraction peaks in the high-angle XRD patterns of ZIF-67@KIT-6 matched with the characteristic ones of the simulated ZIF-67, indicating the presence of small MOF crystals. The weak diffraction intensity might be related to the low content of the MOF crystals that were further covered by the KIT-6 framework. The specific surface areas and porosities of the resultant materials were measured by N_2_ adsorption–desorption experiments (Fig. S1 and Table S1†). The hysteresis loop and steepness of the parent KIT-6 isotherm suggested a typically mesoporous structure.^39^ After ZIF-67 encapsulation, a remarkable decrement in N_2_ adsorption capacity and pore size was observed for ZIF-67@KIT-6, indicating some of the KIT-6 pores were occupied by the encapsulated ZIF-67 crystals.^40^,^41^

Fourier transform infrared spectroscopy (FT-IR) was used to further investigate the composition of ZIF-67@KIT-6 (Fig. S1†). For the parent KIT-6, the two major absorption bands at 1080 and 790 cm^–1^ revealed stretching vibrations of Si–O–Si bonds.^42^

For pure ZIF-67, the band at 425 cm^–1^ was assigned to the vibration of Co–N bonds. Bands at 600–1500 cm^–1^ were attributed to aromatic rings and C–H stretching. The weak band at 1579 cm^–1^ was related to the vibration of C=N bonds.^43^ The presence of all these characteristic bonds for KIT-6 and ZIF-67 confirmed the presence of both KIT-6 and ZIF-67 in the ZIF-67@KIT-6.

**Fig. 1:**
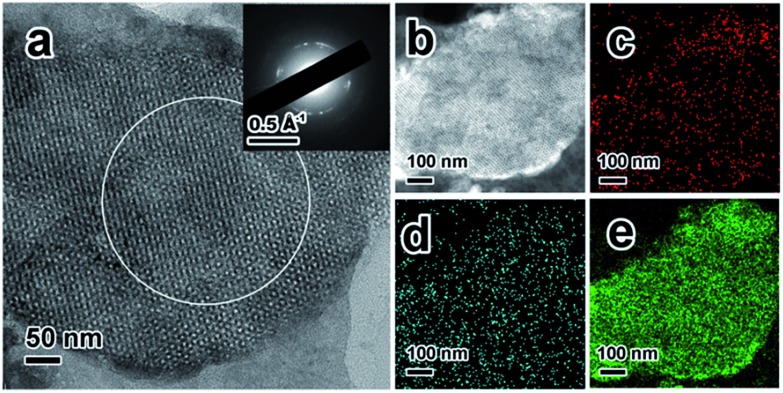
(a) TEM image of ultrathin cuts of ZIF-67@KIT-6, (b) STEM image and corresponding elemental mapping of (c) Co, (d) N and (e) Si elements. Insert in (a): SAED patterns of the selected area (white circle).

To obtain a better understanding of the synthesized ZIF-67@KIT-6, scanning electron microscopy (SEM) and transmission electron microscopy (TEM) analyses were conducted on the particles and ultrathin cuts of the material. SEM and TEM images shown in Fig. S2a and b† revealed that the ZIF-67@KIT-6 materials had smooth external surfaces, on which no ZIF-67 nanoparticles could be observed, indicating a perfect filling of ZIF-67 in the mesopores of KIT-6. As shown in Fig. 1a, the 3D cubic pore structures of KIT-6 were well preserved after the incorporation of MOF. The ring-like selected-area electron diffraction (SAED) patterns pointed out the polycrystalline nature of ZIF-67 on the sample, in which the reciprocal diameter was 0.667 Å^–1^, in good agreement with the simulated results (Fig. S2c†), further confirming the crystalline structure of ZIF-67.^44^ High-angle annular dark-field scanning TEM (HAADF-STEM) images and elemental mappings revealed the even distribution of Co and N elements on the section of the mesoporous material (Fig. 1b–e).

To exclude the possibility of residual uncoordinated Co ions in the ZIF-67@KIT-6, a KIT-6-blank sample was synthesized for comparison without the addition of organic ligands during the synthesis process. After differential centrifugation and thorough washing, atomic adsorption spectroscopy (AAS) was used to quantify the Co content. To our delight, the Co content was below the detection limit, indicating no significant amount of Co residual in KIT-6. Moreover, the Co and C contents of ZIF-67@KIT-6 were also analyzed and showed the molar ratio of Co to C was almost the same as to those of pure ZIF-67 (Table S2†). These results suggest that all Co ions in ZIF-67@KIT-6 would have been assembled into ZIF-67.

**Fig. 2:**
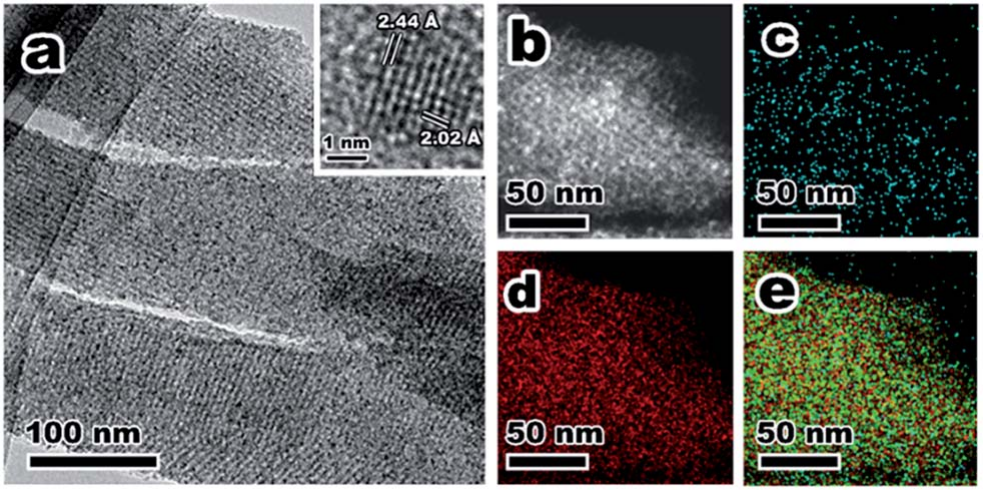
(a) TEM images, (b) STEM, and corresponding elemental mappings of (c) Co, (d) Si and (e) Co + Si + O of Co@KIT-6 ultrathin cuts. Homogeneously dispersed Co_3_O_4_ NPs can be obviously observed in the TEM and STEM images as dark and bright spots, respectively. Inset in (a): HRTEM image of one individual Co_3_O_4_ particle.

ZIF-67@KIT-6 was then calcined under an oxygen atmosphere at 250 °C for 3 h to yield Co@KIT-6. Calcination led to a complete removal of organic ligands of ZIF-67, while the 3D cubic pore structure was well retained (Fig. S1†). Compared with ZIF-67@KIT-6, the Co content in Co@KIT-6 increased from 10.1 wt% to 13.6 wt% (Table S2†) and at the same time, both the BET surface area and pore size were also enhanced (Fig. S1 and Table S1†). In comparison with the parent KIT-6, the slight decrements in N_2_ adsorption capacity and pore size indicated that some pores of KIT-6 were still occupied by Co oxide NPs.^45^–^47^ X-ray photoelectron spectroscopy (XPS) was used to determine the valence state of Co (Fig. S3†). Two peaks at 779.6 and 780.9 eV in the Co 2p_3/2_ region were assigned to Co^2+^ and Co^3+^ species, respectively. A 0.5 atomic ratio of Co^2+^/Co^3+^ indicated the Co ions were mostly converted to Co_3_O_4_ after calcination in O_2_.^48^ From the TEM and STEM images of the ultrathin cuts, Co_3_O_4_ nanoparticles could be obviously observed within the channels of KIT-6 with an average size of *ca.* 2 nm (Fig. 2a and b). The HRTEM image taken from one individual Co_3_O_4_ nanoparticle clearly showed the lattice fringes of 2.02 and 2.44 Å, corresponding to the (400) and (311) facets of Co_3_O_4_, respectively. Elemental mappings confirmed the homogeneous distribution of Co_3_O_4_ on the ultrathin section of Co@KIT-6 (Fig. 2c–e). Despite the good degree of crystallization of Co_3_O_4_, no Co_3_O_4_ phase was detected in the XRD for Co@KIT-6. For comparison, we synthesized a Co/KIT-6 material by a widely employed wetness impregnation method, with the same Co content as Co@KIT-6 (Table S2†). From the TEM images (Fig. S4†), aggregated Co_3_O_4_ NPs with an average size of *ca.* 13 nm could be clearly seen on the outer surface of KIT-6. For Co/KIT-6, the characteristic XRD diffractions of Co_3_O_4_ with high intensities were observed (Fig. S1b†). Thus, we could conclude that the undetected Co_3_O_4_ XRD diffractions in Co@KIT-6 were due to the ultrafine particle sizes.

**Fig. 3:**
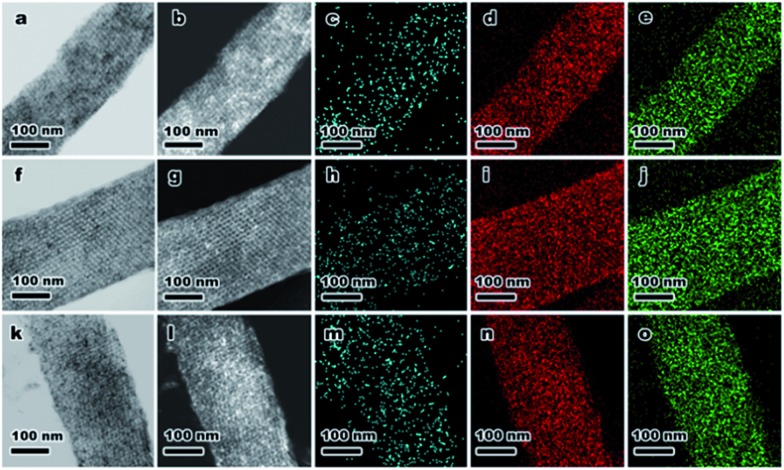
(a–e) TEM, STEM images and corresponding elemental mappings of Fe, Si and O of Fe@KIT-6; (f–j) TEM, STEM images and corresponding elemental mappings of Cu, Si and O of Cu@KIT-6; (k–o) TEM, STEM images and corresponding elemental mappings of Ni, Si and O of Ni@KIT-6.

To demonstrate the versatility of this novel synthetic strategy, other transition-metal NPs encapsulated in KIT-6 materials were also prepared using different MOF precursors, including MIL-88b (Fe), HKUST-1 (Cu) and Ni-ZIF (Fig. S5†). After self-assembly of MOFs in the mesopores of KIT-6, MIL-88b@KIT-6, HKUST-1@KIT-6 and Ni-ZIF@KIT-6 were obtained (Fig. S6–S8†). TEM images and elemental mappings of ultrathin cuts (Fig. 3) gave better illustrations of the metal-oxide distribution within the mesoporous matrix, demonstrating homogeneous distributions of CuO, Fe_3_O_4_ and NiO NPs. HR-TEM images taken from an individual nanoparticle (Fig. S6–S8†) clearly showed lattice fringes of Fe_3_O_4_ (1.64 and 1.71 Å of (511) and (422) facets, respectively), CuO (1.78 Å of (112) facet) and NiO (1.47 Å of (220) facet), which were in good agreement with the XPS results.^48^,^49^

Notably, controlled calcination is crucial to achieve ultrafine metal-oxides NPs, for example, heating rate, calcination temperature and time. A sufficient calcination time was critical for a complete oxidation of metal ions and the removal of organic ligands. In our strategy, the calcination temperature and heating rate were carefully controlled at 250 °C (over the decomposition temperatures of the MOFs in an O_2_ atmosphere^50^–^52^) and 1 °C min^–1^, respectively. According to the Kirkendall effect, ordered and dispersed metal ions tend to aggregate in order to reduce their free surface energy under thermal treatment. At higher temperatures (*e.g.*, 275 and 300 °C), metal ions were more likely to aggregate into larger particles (>5 nm) (Fig. S9a–d†). On the other hand, higher heating rates (2 and 3 °C min^–1^) accelerated the decomposition process, and also led to larger sizes (∼5 nm) (Fig. S9e–h†). Therefore, the key points to obtain ultrafine Co_3_O_4_ NPs were slow heating rates, low calcination temperatures and sufficient thermolysis times.

**Fig. 4:**
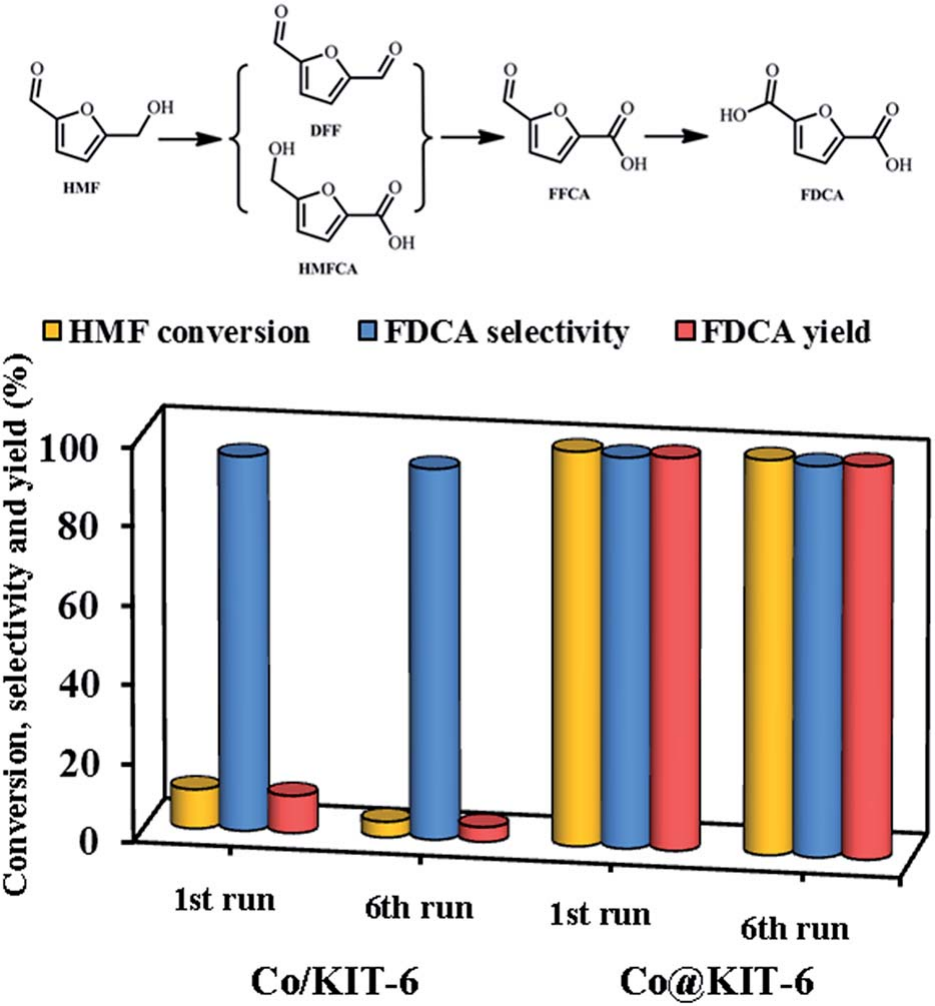
Catalytic performance and reusability tests of Co/KIT-6 and Co@KIT-6 in HMF oxidation.

Finally, the obtained metal-oxide@KIT-6 materials were employed as catalysts for the aerobic oxidation of HMF to FDCA. Among these materials, Co@KIT-6 exhibited the highest reactivity (Table S3†). For comparison, the catalytic activities of KIT-6 and Co/KIT-6 were also investigated. The reaction was carried out at 80 °C using air as the oxidant and water as the solvent. Parent KIT-6 gave no conversion under the reaction condition. The Co/KIT-6 prepared by a typical impregnation method exhibited an unsatisfied activity with only *ca.* 10% HMF conversion and 9.5% FDCA yield (Fig. 4). To our delight, Co@KIT-6 was highly efficient for this transformation under the investigated reaction conditions. As compared to Co/KIT-6, it is remarkable that the FDCA yield was enhanced by a factor of *ca.* 10.5 over Co@KIT-6. Notably, no significant loss in activity or selectivity was observed on Co@KIT-6 even after six runs, while the Co/KIT-6 catalyst deactivated dramatically (Fig. 4). After six reuses, the FDCA yield over the Co@KIT-6 catalyst was 26.6 times higher in comparison to that observed for Co/KIT-6.

Significantly, the developed Co@KIT-6 catalyst also outperformed the previously literature reported noble-metal heterogeneous catalysts, with a remarkable enhancement of *ca.* 3–150 times in TOF under milder, or at least comparable, conditions (Table S3†).^53^–^61^ The superior activity of Co@KIT-6 could be attributed to the presence of ultrafine Co_3_O_4_ NPs confined in the mesopores of KIT-6, which were highly oxidative and thus efficient in oxidation of HMF to FDCA following the Mars–van Krevelen mechanism. According to this mechanism, the lattice oxygen of Co_3_O_4_ NPs facilitated HMF oxidation to FDCA, which was subsequently replenished by molecular oxygen in the air.^62^

We also investigated the effects of reaction temperature, solvent and reaction time on HMF oxidation over the Co@KIT-6 catalyst. HMF conversion increased with increasing the reaction temperature from 60 to 80 °C, maintaining >99% selectivity to FDCA (Table S4†). Meanwhile, H_2_O, in terms of both HMF conversion and FDCA selectivity, was shown to be the best one among the investigated solvents. Under the optimized condition, Co@KIT-6 afforded an almost quantitative conversion of HMF to FDCA at 80 °C within 2 h (Table S4†).

Both Co@KIT-6 and Co/KIT-6 catalysts showed good magnetic properties (Fig. S10a†) and could be easily separated from the reaction mixture by using a simple magnet after the reaction. TG-DSC, TEM, HAADF-STEM and AAS were used to characterize the recycled catalysts. The TG-DSC results indicated that both the Co@KIT-6 and Co/KIT-6 catalysts exhibited good thermal stability even after six cycles (Fig. S10b†). Furthermore, neither obvious aggregation nor significant Co leaching was observed for Co@KIT-6 (Fig. S11 and Table S4†). On the contrary, remarkable Co leaching and serious aggregation of Co_3_O_4_ NPs were detected on the Co/KIT-6 catalyst (Fig. S12 and Table S5†). The significantly improved activity and stability of Co@KIT-6 is believed to be related to the nano-confinement effects of the KIT-6 mesopores, which prevent ultrafine Co_3_O_4_ NPs from aggregating and leaching during the reaction process.

## Conclusions

In summary, we have successfully developed a novel strategy to synthesize ultrafine metal-oxides NPs encapsulated within the mesopores of KIT-6. The synthesis is simple and highly effective, and only involves two steps without the use of costly precursors. The oxide NPs are exclusively confined and uniformly distributed in the mesopores with a high metal loading of up to 13.6 wt%. Benefitting from the encapsulation effects, the as-synthesized Co@KIT-6 materials exhibit superior catalytic performances as compared to conventional supported Co-based catalysts, even heterogeneous noble-metals in HMF oxidation. This strategy could be easily extended to the synthesis of other types of mesoporous silica materials encapsulating various metal NPs, and thus offers a new general approach to prepare novel nanoscale composite materials for advanced applications including catalysis as demonstrated here.

## Conflicts of interest

There are no conflicts of interest to declare.

## Supplementary Material

Supplementary information
