# Supplementary material for: Encapsulation of ultrafine metal-oxide nanoparticles within mesopores for biomass-derived catalytic applications
Source: Chem Sci. 2018 Jan 4;9(7):1854–9. doi: 10.1039/c7sc04724j (PMC5892127; doi:10.1039/c7sc04724j)
Supplement: Supplementary file 1 [file SC-009-C7SC04724J-s001.pdf]

Electronic Supplementary Material (ESI) for Chemical Science.
This journal is © The Royal Society of Chemistry 2018

Supporting Information

Encapsulation of Ultrafine Metal-Oxides Nanoparticles within Mesopores for Biomass-Derived Catalytic Applications

Ruiqi Fang,[a] Panliang Tian,[a] Xianfeng Yang,[b] Rafael Luque,*[c] and Yingwei Li*[a]

[a]*State Key Laboratory of Pulp and Paper Engineering, School of Chemistry and Chemical Engineering, South China University of Technology, Guangzhou 510640, China. Email: liyw@scut.edu.cn*

[b]*Analytical and Testing Centre, South China University of Technology, Guangzhou 510640, China.*

[c]*Departamento de Química Orgánica, Universidad de Córdoba, Edif. Marie Curie, Ctra Nnal IV$_a$, Km 396, E14014, Córdoba, Spain. Email: q62alsor@uco.es*

This PDF file includes:

Materials and methods

Tables S1 to S5

Figures S1 to S12

Experimental

All reagents are of analytical grade and were used without further purification.

Synthesis of ZIF-67

In a typical synthesis, 0.73 g of $Co(NO_3)_2 \cdot 6H_2O$ was dissolved in 15 mL methanol to form a clear solution A. Then, 0.82 g of 2-methylimidazole was dissolved in 15 mL methanol to form a clear solution B. The solutions A and B were mixed together at room temperature and stirred for 24 h. The resulting purple powder was washed with methanol several times and dried under vacuum at 50 ºC overnight.

Synthesis of Co@KIT-6

In a typical synthesis, a 5 mL methanol solution containing $Co(NO_3)_2 \cdot 6H_2O$ (1.02 g) and KIT-6 (1.0 g) was first prepared and treated with under vacuum to remove the adsorbed air. Then, another 5 mL of freshly prepared methanol solution containing 2-methylimidazole (1.15 g) was added under vigorous stirring. The mixture was further stirred for 24 h under vacuum, followed by centrifugation and washing with methanol for several times. The resultant powder was denoted as ZIF-67@KIT-6. After drying at 50 ºC overnight, the ZIF-67@KIT-6 sample was calcined at 250 ºC for 3 h with a heating rate of 1 ºC min^{-1} from room temperature in O_2 atmosphere. The obtained material was denoted as Co@KIT-6.

For comparison, the ZIF-67@KIT-6 samples were subjected to different calcination progresses. The prepared materials were denoted as Co@KIT-6-x, where x indicated the calcination temperatures or heating rates.

Synthesis of Cu@KIT-6

In a typical synthesis, a 5 mL methanol solution containing $Cu(NO_3)_2·3H_2O$ (0.73 g) and KIT-6 (1.0 g) was first prepared and treated under vacuum to remove the adsorbed air. Then, another 5 mL of freshly prepared methanol solution containing 1,3,5-benzenetricarboxylate (0.17 g) was added under vigorous stirring. The mixture was further stirred at 80 ºC for 20 h under vacuum, followed by centrifugation and washing with methanol for several times. The resultant powder was denoted as HKUST-1@KIT-6. After drying at 50 ºC overnight, the HKUST-1@KIT-6 sample was calcined at 250 ºC for 3 h with a heating rate of 1 ºC min^{-1} from room temperature in O_2 atmosphere. The obtained material was denoted as Cu@KIT-6.

Synthesis of Fe@KIT-6

In a typical synthesis, a 5 mL *N,N*-dimethylformamide solution containing $FeCl_3·6H_2O$ (0.81 g) and KIT-6 (1.0 g) was first prepared and treated under vacuum to remove the adsorbed air. Then, another 5 mL of freshly prepared *N,N*-dimethylformamide solution containing terephthalic acid (0.5 g) was added under vigorous stirring. The mixture was further stirred at 100 ºC for 12 h under vacuum, followed by centrifugation and washing with *N,N*-dimethylformamide for several times. The resultant powder was denoted as MIL-88b@KIT-6. After drying at 50 ºC overnight, the MIL-88b@KIT-6 sample was calcined at 250 ºC for 3 h with a heating rate of 1 ºC min^{-1} from room temperature in O_2 atmosphere. The obtained material was denoted as Fe@KIT-6.

Synthesis of Ni@KIT-6

In a typical synthesis, a 5 mL methanol solution containing $Ni(NO_3)_2 \cdot 6H_2O$ (1.02 g) and KIT-6 (1.0 g) was first prepared and treated under vacuum to remove the adsorbed air. Then, another 5 mL of freshly prepared methanol solution containing 2-methylimidazole (1.15 g) was added under vigorous stirring. The mixture was further stirred at 140 ºC for 12 h under vacuum, followed by centrifugation and washing with methanol for several times. The resultant powder was denoted as Ni-ZIF@KIT-6. After drying at 50 ºC overnight, the Ni-ZIF@KIT-6 sample was calcined at 250 ºC for 3 h with a heating rate of 1 ºC min^{-1} from room temperature in O_2 atmosphere. The obtained material was denoted as Ni@KIT-6.

Synthesis of KIT-6-blank

In a typical synthesis, a 5 mL methanol solution containing KIT-6 (1.0 g) was prepared and treated with liquid nitrogen under vacuum to remove the adsorbed air. After that, another 5 mL freshly prepared methanol solution containing $Co(NO_3)_2 \cdot 6H_2O$ (0.73 g) was added under vigorously stirring. The mixture was stirred for 24 h under vacuum followed by centrifugation and washing with methanol for several times, the obtained powder was denoted as KIT-6-blank.

Synthesis of Co/KIT-6

Co/KIT-6 was prepared by a wetness impregnation method. In a typical synthesis, a 5 mL methanol solution containing KIT-6 (1.0 g) was prepared. Then, another 5 mL

freshly prepared methanol solution containing $Co(NO_3)_2 \cdot 6H_2O$ (1.02 g) was added under vigorous stirring. The mixture was further stirred for 24 h followed by centrifugation and drying at 50 ºC overnight. Then the pink powder was calcined at 250 ºC for 3 h with a heating rate of 1 ºC min^{-1} from room temperature in O_2 atmosphere. The obtained material was denoted as Co/KIT-6.

Catalyst characterization

Powder X-ray diffraction (PXRD) patterns of the samples were obtained on a Rigaku diffractometer (D/MAX-IIIA, 3kW) using Cu Kα radiation (40 kV, 30 mA, λ = 0.1543 nm). BET surface areas and pore size measurements were performed with N_2 adsorption/desorption isotherms at 77 K on a Micromeritics ASAP 2020M instrument. Before measurements, samples were degassed at 100 ºC for 12 h. The cobalt contents in the samples were measured quantitatively by atomic absorption spectroscopy (AAS) on a HITACHI Z-2300 instrument. Elemental analysis was performed on an Elementar Vario EL III equipment by weighing samples of 0.2–0.3 mg and packing with aluminum foil for the measurement. Fourier transform infrared spectroscopy (FT-IR) measurements were carried out on a Thermo Fisher iS10 instrument equipped with a liquid nitrogen cooled MCT detector. The magnetic properties of the catalyst were measured on a Quantum Design physical property measurement system (PPMS-9). Thermogravimetric analysis (TG) was carried out on a NETZSCH STA 409 PC/PG under air atmosphere (20 $mL \cdot min^{-1}$).

The size and morphology of materials were studied by scanning electron

microscopy (SEM) and transmission electron microscopy (TEM). SEM was carried out on a Zeiss Merlin instrument. TEM and high-angle annular dark-field scanning transmission electron microscopy (HAADF-STEM) were recorded on a JEOL JEM-2010F instrument equipped with EDX analysis (Bruker XFlash 5030T) operated at 200 kV. In order to better analyze the dispersion and the sizes of the cobalt oxide nanoparticles, the samples were embedded in Spurr's resin and cut into ~30 nm thin sections using the ultramicrotome.

XPS measurements were performed in a ultra-high vacuum (UHV) multipurpose surface analysis system (SpecsTM model Germany) operating at pressures $<10^{-10}$ mbar using a conventional X-ray source (XR-50, Specs, Mg K_{alpha}, 1253.6 eV) in a "stop-and-go" mode to reduce potential damage due to sample irradiation. Binding energies were referenced to the C1s line at 284.6 eV from adventitious carbon. Deconvolution curves for the XPS spectra were obtained using software supplied by the spectrometer manufacturer.

General procedures for HMF oxidation

In a typical run, HMF (1.0 mmol) and catalyst (metal 1 mol%) were added to H_2O (3 mL) in the autoclave with magnetic stirrer. After purging the reactor several times with air, the reactor was maintained at 80 ºC, 0.1 MPa air for 2 h with a stirring speed of 600 rpm. During the reaction, samples were taken and analyzed by HPLC (Shimazu LC-20A). The first sample was taken when the reaction temperature reached 80 ºC. After reaction, the reactor was cooled to room temperature and the catalyst was isolated from the solution by centrifugation, washed with methanol and

reused directly.

The conversion of HMF, selectivity and yield of FDCA are calculated as follows:

$$\text{HMF conversion} = \left(1 - \frac{\text{Moles of HMF}}{\text{Moles of HMF loaded}}\right) \times 100\%$$

$$\text{FDCA selectivity} = \frac{\text{Moles of FDCA}}{\text{Moles of } HMF \text{ converted}} \times 100\%$$

$$\text{FDCA yield} = \frac{\text{Moles of FDCA}}{\text{Moles of } HMF \text{ loaded}} \times 100\%$$

$$\text{Turnover frequency} = \frac{\textit{Moles of HMF converted}}{\textit{Moles of active sites}} \times \frac{1}{\textit{reaction time}}$$

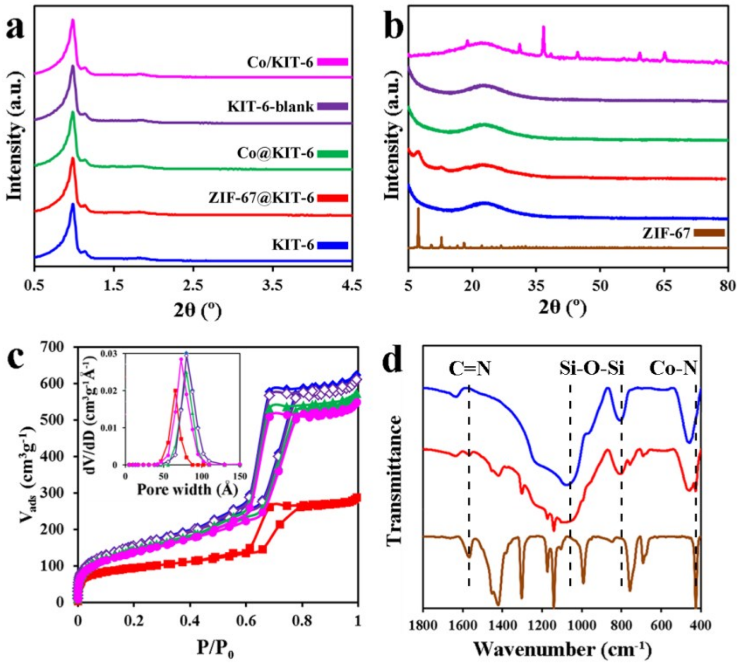

Fig. S1 (a) Low-angle and (b) high-angle powder XRD patterns, (c) nitrogen adsorption/desorption isotherms and pore size distribution and (d) FT-IR spectra of Co-based materials.

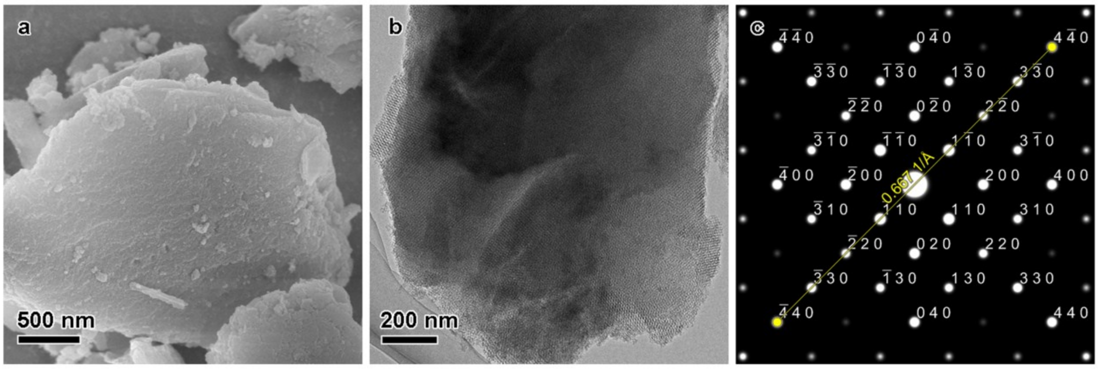

Fig. S2 (a) SEM and (b) TEM images of the ZIF-67@KIT-6 material, (c) simulated FFT patterns of the ZIF-67 crystal. The reciprocal distance between two (440) spots is 0.667 $Å^{-1}$, revealing that the lattice distance of (440) planes for the ZIF-67 crystal is 2.99 Å.

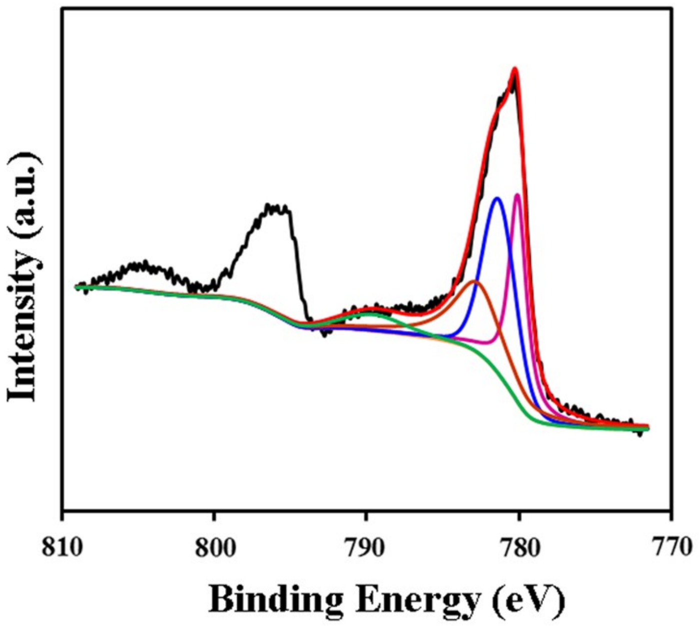

Fig. S3 XPS patterns of the Co 2p region of Co@KIT-6.

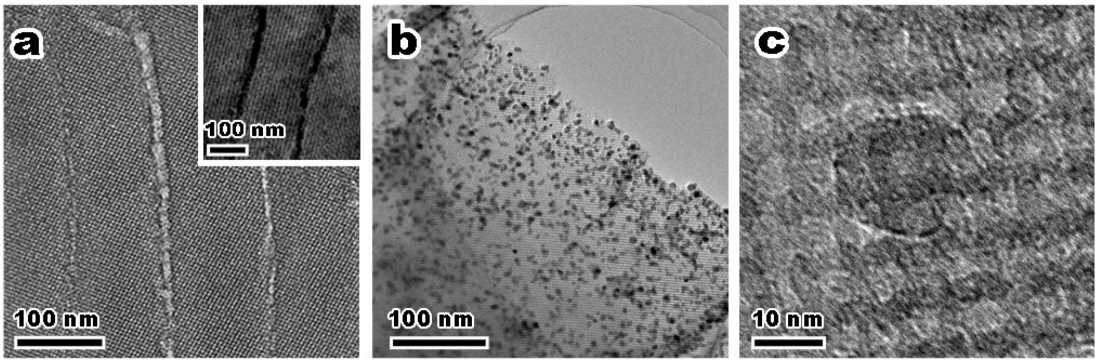

Fig. S4 TEM images of (a) parent KIT-6 ultrathin cuts, (b) Co/KIT-6 and (c) HRTEM of an individual Co_3O_4 nanoparticle.

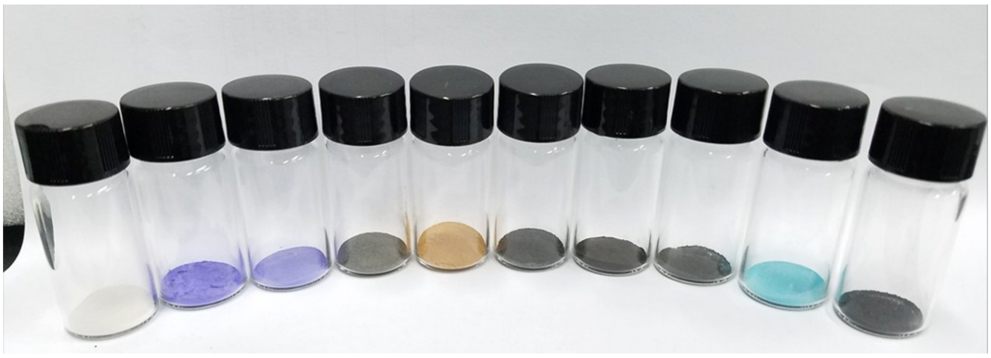

Fig. S5 The photographs of the as-prepared nanocomposites. From left to right: KIT-6, ZIF-67, ZIF-67@KIT-6, Co@KIT-6, MIL-88b@KIT-6, Fe@KIT-6, Ni-ZIF@KIT-6, Ni@KIT-6, HKUST-1@KIT-6, and Cu@KIT-6.

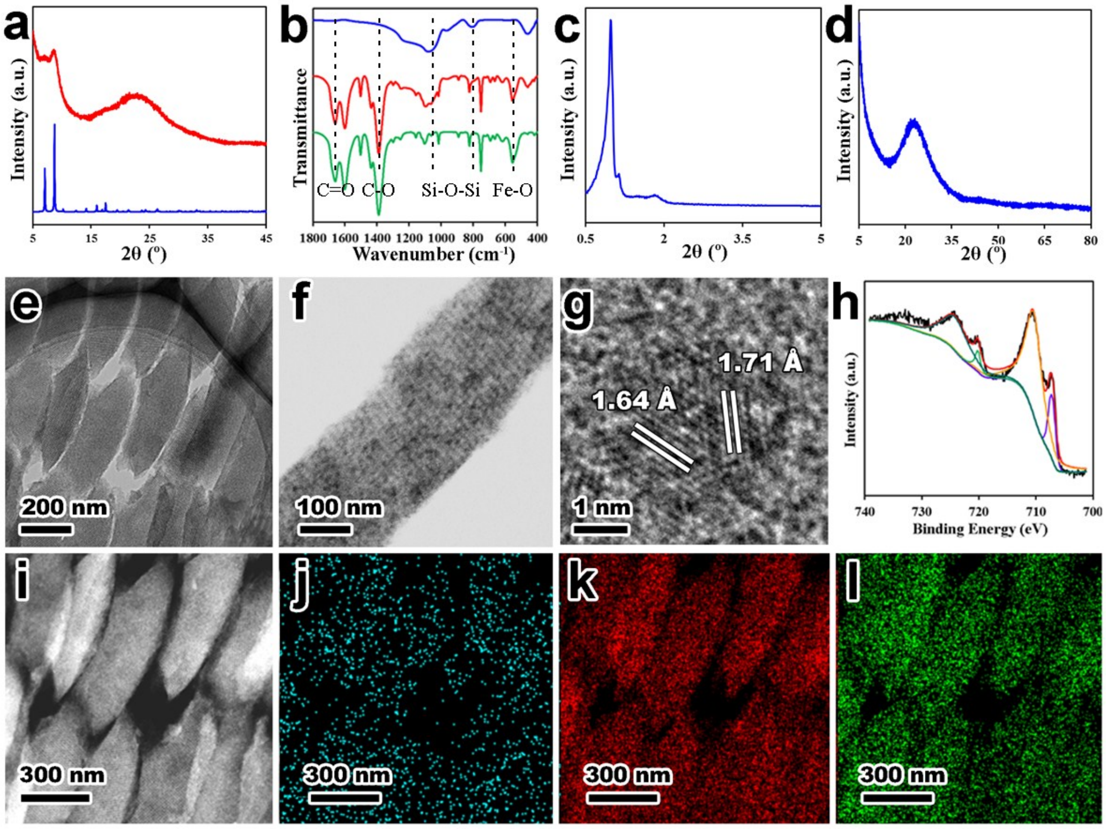

Fig. S6 (a) XRD patterns of MIL-88b@KIT-6 and simulated MIL-88b, (b) FT-IR spectra of KIT-6, MIL-88b@KIT-6 and MIL-88b (from top to bottom. For the parent KIT-6, the two major absorption bands at 1080 and 790 cm^{-1} revealed stretching vibrations of Si-O-Si bonds. For pure MIL-88b, the band at 550 cm^{-1} was assigned to the vibration of Fe-O bonds. The strong bands at 1399 and 1663 cm^{-1} were related to the vibration of C-O and C=O bonds), (c, d) Low- and high-angle XRD patterns of Fe@KIT-6, (e, f) TEM images of Fe@KIT-6 ultrathin cuts, in which homogeneously dispersed Fe_3O_4 NPs could be obviously observed as dark spots, (g) HR-TEM image of an individual Fe_3O_4 nanoparticle, (h) XPS patterns of Fe 2p region, (i-l) STEM image and elemental mappings of Fe, Si and O of Fe@KIT-6 ultrathin cuts.

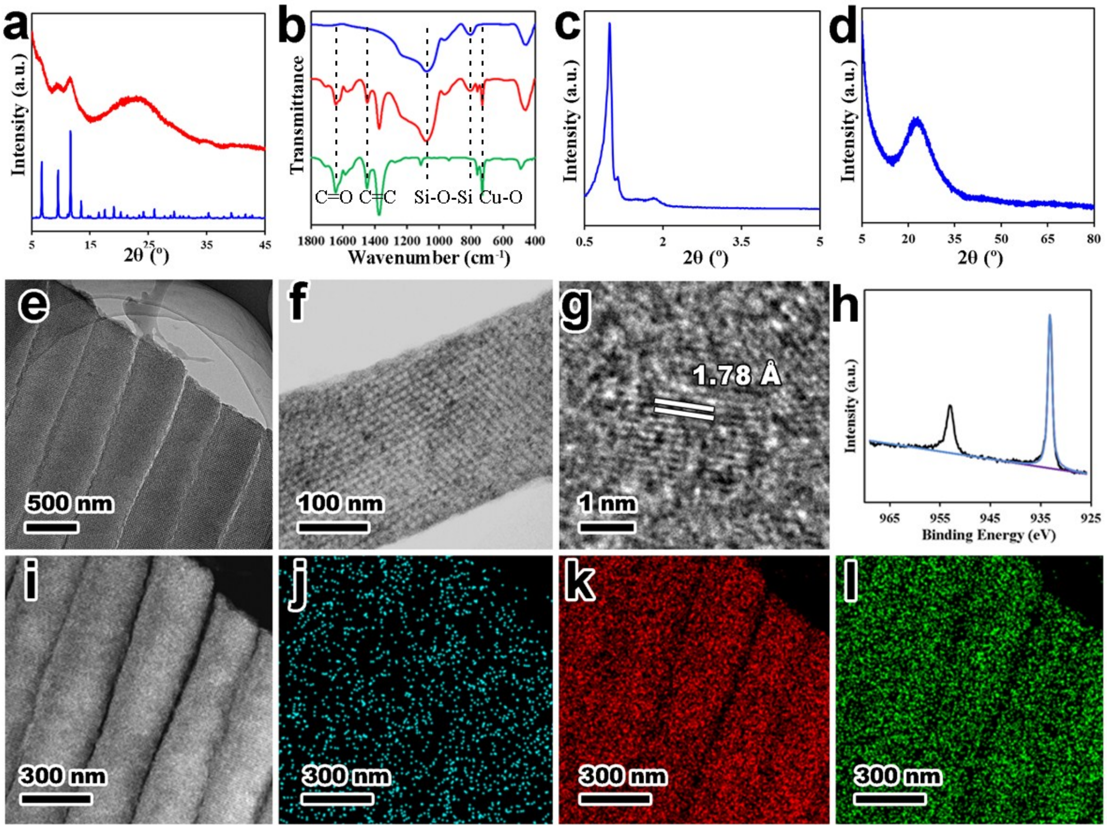

Fig. S7 XRD patterns of HKUST-1@KIT-6 and simulated HKUST-1, (b) FT-IR spectra of KIT-6, HKUST-1@KIT-6 and HKUST-1 (from top to bottom. For the parent KIT-6, the two major absorption bands at 1080 and 790 cm^{-1} revealed stretching vibrations of Si-O-Si bonds. For pure HKUST-1, the band at 620 cm^{-1} was assigned to the vibration of Cu-O bonds. The strong bands at 1449 and 1647 cm^{-1} were related to the vibration of C=C and C=O bonds), (c, d) Low- and high-angle XRD patterns of Cu@KIT-6, (e, f) TEM images of Cu@KIT-6 ultrathin cuts, in which homogeneously dispersed CuO naoparticles could be obviously observed as dark spots, (g) HR-TEM image of an individual CuO nanoparticle, (h) XPS patterns of Cu 2p region, (i-l) STEM image and elemental mappings of Cu, Si and O of Cu@KIT-6 ultrathin cuts.

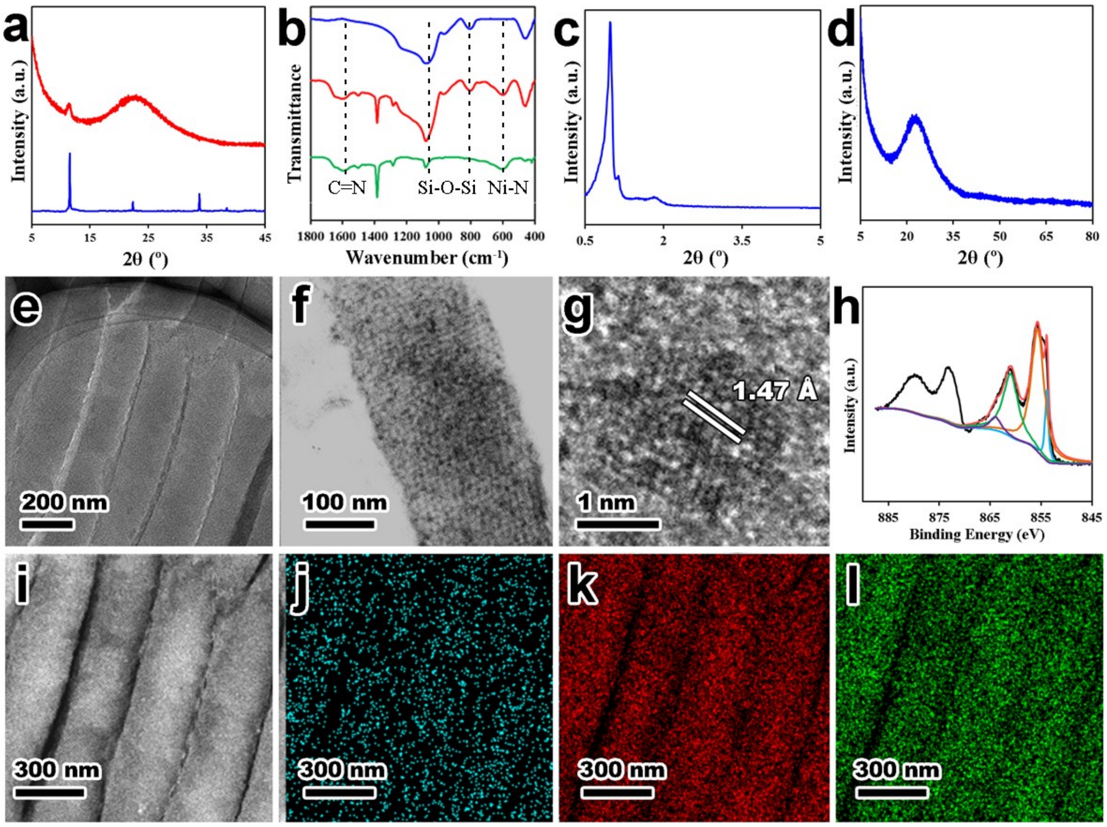

Fig. S8 XRD patterns of Ni-ZIF@KIT-6 and Ni-ZIF, (b) FT-IR spectra of KIT-6, Ni-ZIF@KIT-6 and Ni-ZIF (from top to bottom. For the parent KIT-6, the two major absorption bands at 1080 and 790 cm^{-1} revealed stretching vibrations of Si-O-Si bonds. For pure Ni-ZIF, the band at 598 cm^{-1} was assigned to the vibration of Ni-N bonds. The weak band at 1590 was related to the vibration of C=N bond), (c, d) Low- and high-angle XRD patterns of Ni@KIT-6, (e, f) TEM images of Ni@KIT-6 ultrathin cuts, in which homogeneously dispersed NiO naoparticles could be obviously observed as dark spots, (g) HR-TEM image of an individual NiO nanoparticle, (h) XPS patterns of Ni 2p region, (i-l) STEM image and elemental mappings of Ni, Si and O of Ni@KIT-6 ultrathin cuts.

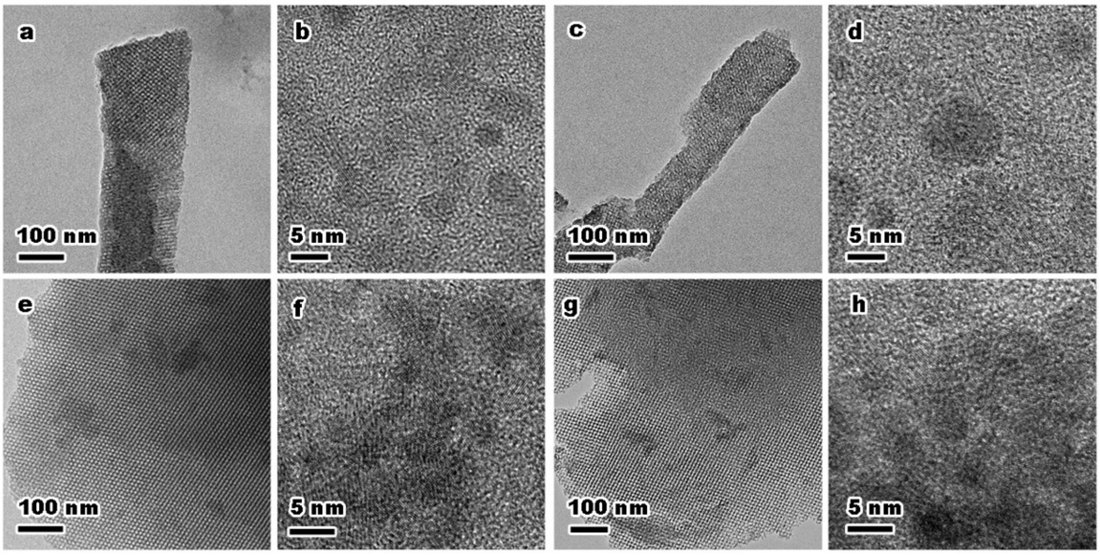

Fig. S9 TEM images of (a, b) Co@KIT-6-275, (c, d) Co@KIT-6-300, (e, f) Co@KIT-6-2 and (g, h) Co@KIT-6-3.

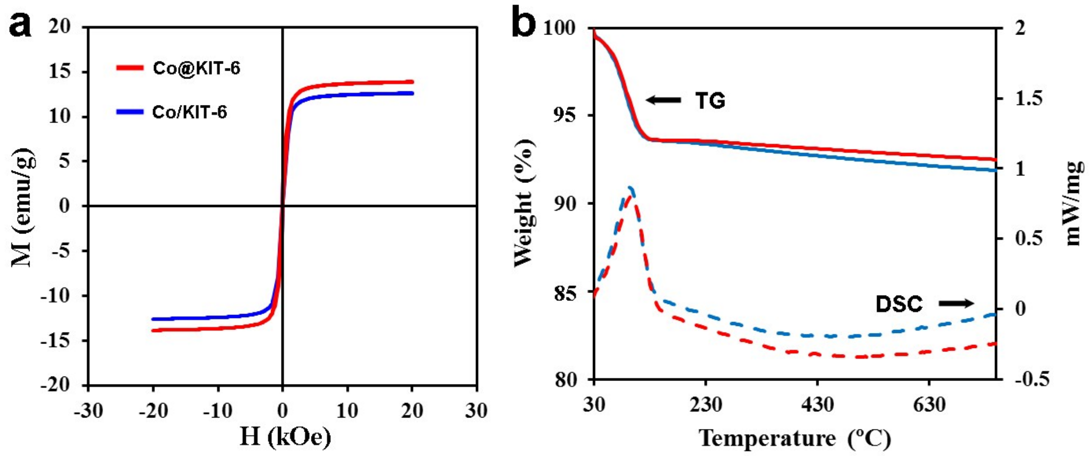

Fig. S10 (a) Hysteresis loops of Co@KIT-6 and Co/KIT-6, and (b) TG-DSC curves of the recycled Co@KIT-6 (red) and Co/KIT-6 (blue) catalysts.

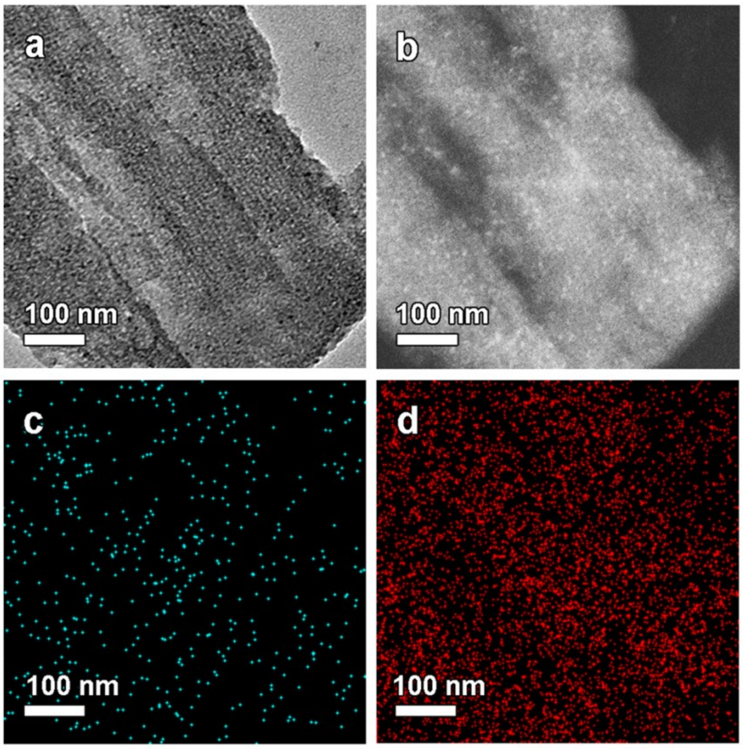

Fig. S11 TEM image (a), STEM image (b), and elemental mappings of Co (c) and Si (d) on ultrathin cuts of the Co@KIT-6 catalyst after six runs.

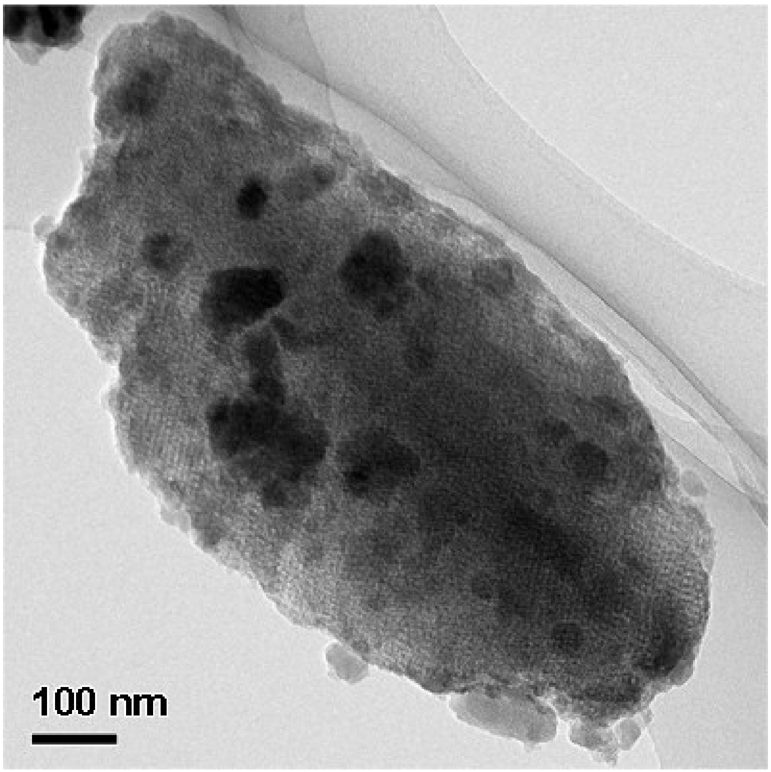

Fig. S12 TEM image of the Co/KIT-6 catalyst after six runs.

Table S1. Surface areas and pore volumes of the obtained materials.

Sample	S_{BET} ($m^2\ g^{-1}$)	$S_{Langmuir}$ ($m^2\ g^{-1}$)	V_{pore} ($cm^3\ g^{-1}$)	D_{pore} (nm)
ZIF-67@KIT-6	301	425	0.40	6.1
Co@KIT-6	522	694	0.58	7.3
Co/KIT-6	550	734	0.66	7.0
KIT-6-blank	602	780	0.70	7.7
KIT-6	608	781	0.70	7.8
MIL-88b@KIT-6	326	449	0.42	6.3
Fe@KIT-6	531	701	0.56	7.1
HKUST-1@KIT-6	342	467	0.46	6.2
Cu@KIT-6	539	716	0.58	7.0
NiZIF@KIT-6	335	451	0.41	6.1
Ni@KIT-6	533	708	0.57	7.2

Table S2. Elemental compositions of the as-synthesized materials measured by AAS and elemental analysis.

Sample	Element content (%)				
	C	Co	Fe	Cu	Ni
ZIF-67	43.4	26.7	-	-	-
ZIF-67@KIT-6	16.5	10.1	-	-	-
Co@KIT-6	-	13.6	-	-	-
Co/KIT-6	-	13.6	-	-	-
KIT-6-blank	-	-	-	-	-
MIL-88b@KIT-6	3.4	-	10.7	-	-
Fe@KIT-6	-	-	11.3	-	-
HKUST-1@KIT-6	10.8	-	-	9.4	-
Cu@KIT-6	-	-	-	11.6	-
NiZIF@KIT-6	16.9	-	-	-	10.5
Ni@KIT-6	-	-	-	-	13.4

Table S3. Comparison of results in HMF oxidation to FDCA over different catalysts.[a]

Ref.	Catalyst	T (ºC), atmosphere	Time (h)	Con. (%)	Yield (%)	TOF (h^{-1})
This work	Co@KIT-6	80, 0.1 MPa air	2	100	>99	150
	Fe@KIT-6	95, 0.1 MPa air	2	90	89	135
	Cu@KIT-6	110, 0.1 MPa air	2	87	82	131
	Ni@KIT-6	100, 0.1 MPa air	2	85	80	128
53	AuPd/ZOC	80, 0.3 MPa O_2	4	>99	>99	49.8
58	Au/CeO_2	65, 1 MPa air	48	100	>99	18.75
	Au/TiO_2	65, 1 MPa air	8	100	>99	18.75
59	Au/HY	60, 0.3 MPa O_2	6	>99	>99	18.3
55	Pt/CNTs	95, 0.5 MPa O_2	14	100	98	7.14
61	γFe_2O_3@HAP-Pd	100, O_2 flow (30 mL·min^{-1})	6	97	92.9	6.54
60	Au/HT	65, O_2 flow (50 mL·min^{-1})	7	>99	>99	5.70
54	Pt@PMO-IL-2	80, 0.1 MPa O_2	24	>99	>99	4.17
56	Pt/C-O-Mg	110, 1.0 MPa O_2	6	100	97	4.04
57	Ru/C	120, 0.2 MPa O_2	5	100	88	1.00

[a]TOF = $n_{HMF\ converted}/(n_{active\ sites}\cdot time)$

Table S4. HMF oxidation over Co@KIT-6.

Entry	Temperature (ºC)	Solvent	Time (h)	HMF conversion (%)	FDCA selectivity (%)	FDCA yield (%)
1	60	H_2O	2	63	>99	62
2	70	H_2O	2	85	>99	84
3	80	H_2O	1	52	>99	51
4	80	H_2O	1.5	76	>99	75
5	80	H_2O	2	100	>99	>99
6	80	Toluene	2	75	92	69
7	80	Ethanol	2	83	90	75
8	80	Cyclohexane	2	52	83	43

Reaction conditions: HMF (1.0 mmol), catalyst (metal, 1 mol%), air (0.1 MPa), solvent (3 mL).

Table S5. The Co contents before and after the reaction.

Sample	Co content before reaction (wt%)	Co content after reaction (wt%)	Amount of Co leached (wt%)	Leaching percentage (%)
Co@KIT-6	13.6	13.5	0.1	<1
Co/KIT-6	13.6	12.5	1.1	8.1
